# Psychiatric Diagnoses and Treatment Preceding Schizophrenia in Adolescents Aged 9–17 Years

**DOI:** 10.3389/fpsyt.2020.00487

**Published:** 2020-06-04

**Authors:** Christina D. Kang-Yi, Brian Chao, Shelly Teng, Jill Locke, David S. Mandell, Yin-Ling Irene Wong, C. Neill Epperson

**Affiliations:** ^1^Department of Psychiatry, Perelman School of Medicine, University of Pennsylvania, Philadelphia, PA, United States; ^2^The Leonard Davis Institute of Health Economics, University of Pennsylvania, Philadelphia, PA, United States; ^3^Department of Education, Loyola Marymount University, Los Angeles, CA, United States; ^4^Icahn School of Medicine at Mount Sinai, New York, NY, United States; ^5^Speech and Hearing Sciences, University of Washington, Seattle, WA, United States; ^6^School of Social Policy and Practice, University of Pennsylvania, Philadelphia, PA, United States; ^7^Department of Psychiatry, School of Medicine, University of Colorado, Denver, CO, United States

**Keywords:** schizophrenia, preceding psychiatric diagnoses, preceding psychiatric treatment, adolescents aged 9–17 years, health claims data

## Abstract

**Objective:**

Our study aimed to examine psychiatric diagnoses and treatment preceding a schizophrenia diagnosis in adolescents, stratified by sex and race/ethnicity.

**Methods:**

Using Medicaid physical and behavioral health and pharmacy claims data, we identified 1,459 adolescents who were aged 9–17 years and diagnosed with schizophrenia between January 2006 through June 2009. Psychiatric diagnosis, mental health service use including psychiatric hospitalization, residential treatment and outpatient therapy and psychotropic medication use preceding schizophrenia were identified.

**Results:**

Forty-five percent of the adolescents were diagnosed with one or more psychiatric conditions. More than 40% of the adolescents were hospitalized or placed in a residential treatment facility for other psychiatric conditions preceding schizophrenia. Overall, 72% of the adolescents were prescribed with one or more psychotropic medications and 22% were prescribed with three or more psychotropic medications in the year prior to their first schizophrenia diagnosis. We found that sex and race/ethnicity influence preceding psychiatric conditions and psychiatric treatment use.

**Conclusions:**

Careful screening and evaluation to validate diagnoses is important as the presence of certain psychiatric morbidity is common among adolescents with schizophrenia during the prodromal period. Developing acceptable and accessible interventions that will reduce psychiatric hospitalization and residential treatment care and improve care connection for schizophrenia treatment is important to mitigate complexity in treatment for adolescents and reduce cost burden for families and the society. Integrating health claims data in the development of schizophrenia risk conversion models can be useful in effectively predicting ideal timing of tailored interventions for adolescents with preceding psychiatric conditions.

## Introduction

Many individuals who develop schizophrenia exhibit a gradual increasing rate of psychiatric symptoms preceding psychosis onset. This prodromal stage can span months to years prior to a schizophrenia diagnosis (1). During the prodromal period, genetic and environmental risk factors, such as substance abuse, often are linked with increased risk of psychosis later on (2, 3). The prodromal symptoms are often characterized by symptoms consistent with other psychiatric disorders, thereby leading to early contact with mental health services (4). Previous evidence from birth cohort studies identifies increased risk for schizophrenia among those who have been diagnosed in childhood with conduct disorder, oppositional defiant disorder (ODD), depression, anxiety disorder, or attention-deficit/hyper activity disorder (ADHD) (5).

Examining whether psychiatric conditions have been diagnosed prior to a diagnosis of schizophrenia has two important implications. First, psychiatric symptoms that are common among adolescents (6) may complicate early detection and treatment. About half of individuals with schizophrenia have comorbid psychiatric disorders (7), most commonly depression (8). Schizophrenia symptoms may be misinterpreted in the presence of other symptoms and result in inappropriate labeling and treatment. For example, one study found that patients identified as high risk based on prodromal symptoms were enrolled in treatments like psychotherapy and pharmacotherapy and given medications including antidepressants, antipsychotics, stimulants, and mood stabilizers (9). If these treatments are provided under inappropriate labeling, this delays treatment for psychosis, namely schizophrenia, when diagnosis is determined later on.

Second, early diagnosis and treatment initiation can result in much better outcomes than when diagnosis is delayed (10). The first five years after schizophrenia diagnosis is the stage of maximum risk for treatment disengagement, relapse, and suicide. At the same time, adolescents go through major developmental challenges such as forming a stable identity, peer networks, and intimate relationships, and obtaining vocational training (11). If the presence of certain psychiatric morbidity is common among adolescents with schizophrenia during the prodromal period, it can act as a red flag that leads to more careful screening and evaluation. Early onset schizophrenia in adolescents has substantial loading of externalizing behavior disorders, such as mood/emotional dysregulation disorders, attention deficit disorders, and oppositional defiant/conduct disorder (12). Given that the age of first diagnosis and patterns of other behavioral comorbidities are important factors that differentiate early onset schizophrenia populations (13), identifying comprehensive patterns of comorbid psychiatric disorders and treatments in adolescents can produce more effective outcomes by enabling clinicians to better stratify treatment interventions.

Preceding psychiatric conditions may differ by sex and race/ethnicity. Recent epidemiological studies have indicated that there is a higher incidence of schizophrenia in males compared to females (14). A higher proportion of African Americans than whites are diagnosed with conduct disorder and ODD (15). A higher proportion of whites than other race/ethnic groups are diagnosed with ADHD, bipolar disorder, and autism (15–17). Post-traumatic stress disorder (PTSD) is a more common diagnosis among females than males (18).

Empirical studies on first episode of psychosis and schizophrenia have focused on predictors of symptom remission and functional recovery (19–21). Coordinated specialty care interventions such as the Recovery After an Initial Schizophrenia Episode (RAISE) and the Early Detection and Intervention for Prevention of Psychosis Program (EDIPP) have proven the positive effect of early detection, outreach for treatment engagement and early treatment (21–24), suggesting that early identification of adolescents with schizophrenia is critical. These studies have found that baseline symptom severity, time in treatment and decrease or remission of substance use are key predictors of symptomatic and functional outcome of psychosis and schizophrenia treatment (19–21).

Despite the well-established line of research on first episode of and early intervention for psychosis and schizophrenia, there is significant lack of insights into psychiatric diagnoses and treatment that adolescents go through preceding a schizophrenia diagnosis. Research findings on psychiatric diagnosis and treatment prior to schizophrenia in adolescents can inform effective schizophrenia care delivery. Our study examined psychiatric diagnoses and treatment including both mental health service and psychotropic medication use preceding a schizophrenia diagnosis in adolescents. We stratified the analysis by sex and race/ethnicity based on the previous research finding that psychiatric diagnoses and treatment can differ based on sex and race/ethnicity.

## Methods

Data came from the Pennsylvania behavioral and physical health and pharmacy claims. Adolescents aged 9–17 years with at least two claims with a primary diagnosis of schizophrenia or psychotic disorder (19) between January 1, 2006 through June 30, 2009 were included in the study sample. The Pennsylvania Medicaid data we could use were available only for this duration. International Classification of Disease (9^th^ ed., ICD-9) codes including 295xx, 297.1, 297.3, 298.1, 298.3, 298.4, 298.8, 298.9, and 301.22 were identified as having schizophrenia. The first dates identified for the ICD-9 codes for psychotic disorders or schizophrenia were indexed as the initial schizophrenia diagnosis date. Considering that individuals who receive the first diagnosis of any psychotic disorder subsequently receive a schizophrenia diagnosis, the ICD-9 codes for psychotic disorder were included in identifying the index date for schizophrenia diagnosis. A total of 1,459 adolescents aged 9–17 years with a schizophrenia diagnosis and continuous Medicaid enrollment for one-year prior to the first schizophrenia diagnosis were included in the study sample. The University of Pennsylvania's Institutional Review Board approved this study and the Pennsylvania State Office of Mental Health and Substance Abuse Services approved the use of Medicaid claims data for this study.

Adolescents' ages were measured at the index date of first schizophrenia diagnosis. Race/ethnicity categories included African American, Hispanic, white, and other. Sex abstracted from the claims data as well was measured as male or female. More than one-half of the sample (57%) was male and aged 15–17 years at their first schizophrenia diagnosis (58.5%). The largest racial/ethnic group was African American (44.7%), followed by white (38.2%), Hispanic (14.8%) and other racial/ethnic group (2.4%). The distribution of sex and age did not significantly differ by race/ethnicity.

Psychiatric diagnoses from ICD-9 codes associated with claims during one year prior to the first schizophrenia diagnosis were identified as preceding diagnoses. Diagnoses included bipolar disorder, depression, substance use disorder, post-traumatic stress disorder (PTSD), other mood disorder, adjustment disorder, attention deficit hyperactivity disorder (ADHD), conduct disorder/oppositional defiant disorder (conduct/ODD), autism, and anxiety disorder.

Mental health service use including outpatient therapy, inpatient hospitalization, and residential treatment care was identified through the current procedural technology (CPT) codes and the specialty codes available through the Medicaid claims data. Wraparound services for children were categorized as one of outpatient therapies. Other mental health service use including partial treatment, drug and alcohol treatment and mental health crisis service were excluded from the analysis as these treatment types were associated with only 7.2% of total claims matched for the study sample. The percentage of these mental health service use was minimal at the person-level, and was not meaningful to compare by subgroups. Psychotropic medication classes included: antidepressants, antipsychotics, benzodiazepine, central nervous system (CNS) stimulants, epileptic mood stabilizers, and other sedation. We created a separate variable named as “epileptic mood stabilizers” which included valproic acid, carbamazepine and lithium to distinguish the possible prodromal stage of schizophrenia from other sedation use. These active ingredients are known to be prescribed prior to schizophrenia in adolescents (25, 26).

Statistical analyses were conducted using SAS 9.4 (27). Percentages of psychiatric diagnoses, mental health service use and psychotropic medication use, and mean number of days prescribed for psychotropic medication were calculated for each intersectional sex and race/ethnic group (African American male, African American female, Hispanic male, Hispanic female, White male, White female, males in other race/ethnicity, and females in other race/ethnicity). The other race/ethnicity recoded in Medicaid claims included Asian, American Indian/Alaskan Native and other race/ethnicity, and Asians were the majority of this group included in the study.

Generalized linear models (GLMs) were used to test whether the following dependent variables differ by sex and race/ethnicity: presence of preceding psychiatric diagnoses including ADHD, adjustment disorder, anxiety disorder, autism, bipolar disorder, conduct/ODD, depression, other mood disorder, PTSD, and substance use disorder measured as binary variables (Yes vs. No); total number of preceding psychiatric diagnoses measured as a categorical variable (none, 1, 2, and 3 or more diagnoses); use of psychiatric outpatient therapy, psychiatric hospitalization and psychiatric residential treatment measured as binary variables (Yes vs. No); use of antidepressant, antipsychotic, benzodiazepine, CNS stimulants, epileptic mood stabilizers, and other sedation measured as binary variables (Yes vs. No) and as mean number of days prescribed with each medication; and total number of psychotropic medications used (a categorical variable of none, 1, 2, and 3 or more medication classes). Sidak correction (28) to adjust inequality for all main-effect means for the multiple comparisons was used in the GLMs.

## Results

### Preceding Psychiatric Diagnoses

Forty-five percent of the adolescents were diagnosed with one or more psychiatric conditions (see **Table 1**). Significantly higher percentage of white females was diagnosed with bipolar disorder than were African American and Hispanic males. Hispanic males had the lowest percentage of those diagnosed with bipolar disorder (3.1%, p < .0001). Significantly higher percentages of African American males (32.5%) and African American females (28.3%) were diagnosed with conduct/ODD than were all other groups except other racial/ethnic males and females (p < .0001). Hispanic males had the lowest percentage of those diagnosed with depression (0.8%) and this was significantly lower than that for Hispanic females and white females (11.4% and 9.1%, respectively, p < .01). The percentages of Hispanic females (5.7%) and African American females (4.6%) diagnosed with PTSD were more than double the percentages of all other adolescent groups diagnosed with PTSD. The difference was not statistically significant, but it was approaching the significance level with p = 0.051. Other racial/ethnic male group had the highest percentage of those diagnosed with autism (10% vs. 0%–3.8% for all other groups at p < .0001) and those diagnosed with anxiety disorder (5% vs. 0%–0.3% for all other groups at p < .0001).

**Table 1:** Psychiatric diagnosis and mental health service use preceding schizophrenia diagnosis.

	African American female (N = 283)	African American male (N = 369)	Hispanic female (N = 88)	Hispanic male (N = 128)	White female (N = 243)	White male (N = 314)	Other ethnic female (N = 14)	Other ethnic male (N = 20)
Psychiatric diagnosis	%	%	%	%	%	%	%	%
Bipolar disorder (p < .0001)	12.0	6.5^a^	10.2	3.1^b^	20.2^a,b,c^	11.8^c^	14.3	20.0
Depression (p < .01)	7.8	3.8	11.4^a^	0.8^a,b^	9.1^b^	6.1	21.4	5.0
Substance use disorder	0.7	0.8	0	0	2.5	1.3	0	0
PTSD	4.6	0.5	5.7	0	2.1	1.3	0	0
Other mood disorder (p < .05)	15.2	10.3	13.6	5.5^a^	17.7^a^	14.0	7.1	10.0
Adjustment disorder (p < .01)	2.1^a^	1.6^a^	0.0^a^	0.0^a^	1.2^a^	1.6^a^	14.3^a,b^	0 ^b^
ADHD	4.6	10.8	5.7	10.2	4.5	11.5	7.1	15.0
Conduct/ODD (p < .0001)	28.3^a^	32.5^b^	12.5^a,b^	14.8^b^	12.6^a,b^	17.5^a,b^	7.1	35.0
Autism (p < .0001)	0.4^a^	0.5^b^	0^c^	0^d^	1.2^e^	3.8^a,b,d^	0	10.0^a,b,c,d,e^
Anxiety disorder (p < .0001)	0^a^	0.3^a^	0^a^	0^a^	0^a^	0^a^	0^a^	5.0^a^
Number of psychiatric diagnoses (p < .001)								
None	47.4	52.9	64.8	72.7	54.3	54.5	57.1	35.0
1	35.3	29.5	19.3	21.1	26.8	28.0	28.6	45.0
2	12.7	14.6	9.1	5.5	14.8	13.1	7.1	10.0
3 or more diagnoses	4.6	3.0	6.8	0.8	4.1	4.5	7.1	10.0
Mental health service use								
Outpatient therapy (p < .0001)	2.1^a^	1.6^a,b^	1.1^a^	0.8^a^	7.0^b^	9.6^a^	0	15.0
Inpatient hospitalization (p < .01)	29.7^a^	22.8	29.6	12.5^a^	25.1	19.8	28.6	30.0
Residential treatment care(p < .0001)	33.6^a^	34.7^b^	14.8^a,b^	16.4^a,b^	21.4^a,b^	21.3^a,b^	21.4	40.0

Generalized Linear Models (GLMs) were used to identify significant differences in preceding psychiatric diagnoses and mental health service use. All variables were measured as binary variables (yes vs. no) except the number of psychiatric diagnoses. Sidak correction to adjust for the multiple comparisons was used in the GLMs. Significant differences by intersectional sex and race/ethnicity are presented in the parentheses if p < .05. Pairwise significance between intersectional sex and race/ethnicity are noted with small letters. The same letter indicates significant difference between the intersectional groups at p < .05.

### Mental Health Service Use

More than 40% of the adolescents were hospitalized or placed in a residential treatment facility for psychiatric conditions (see **Table 1**). Higher percentages (about 30%) of African American females, Hispanic females, and both males and females in other racial/ethnic group compared to all other groups were hospitalized for psychiatric conditions in the year prior to their first schizophrenia diagnosis. Of the groups, the percentage of African American females with psychiatric hospitalization was significantly higher than that for Hispanic males (29.7% vs. 12.5%, p < .01). Forty percent of males in other racial/ethnic group and more than one third of African American females and males were placed in residential treatment facilities, while 21% of white females and males and 14%–16% of Hispanic females and males were placed in residential treatment facilities. The residential treatment use significantly differed between whites and other groups except other race/ethnic females and males (p < .0001). Seven percent of white females and 9.6% of white males and 15% of males in other racial/ethnic group received outpatient therapy for one year prior to their first schizophrenia diagnosis, while only 0.8%–2.1% of all other adolescent groups received outpatient therapy. The outpatient therapy use significantly differed between whites and other groups except other racial/ethnic males and females (p < .0001).

### Psychotropic Medication Use

Overall, 71.9% of the adolescents used one or more classes of psychotropic medications and 21.6% were prescribed medications from three or more classes in the year prior to their first schizophrenia diagnosis. As shown in **Table 2**, more than 30% of white females and males and about 25% of Hispanic females and males were prescribed with three or more psychotropic medication classes. The difference in the three or more psychotropic medication use was more prevalent by race/ethnicity than by sex. African American females and males had the lowest percentages (12.4% and 12.7%, respectively), and 32% of white adolescents, 24%–25% of Hispanic females and males, and 14.3% of females and 20% of males in other racial/ethnic group were prescribed with three or more psychotropic medication classes (p < .0001).

**Table 2:** Psychotropic medication use preceding schizophrenia diagnosis.

	African American female (N = 283)	African American male (N = 369)	Hispanic female (N = 88)	Hispanic male (N = 128)	White female (N = 243)	White male (N = 314)	Other ethnic female (N = 14)	Other ethnic male (N = 20)
Percentage of adolescents with psychotropic medication use	%							
Antidepressant (p < .0001)	27.9^a^	20.6^b^	48.9 ^b^	29.7^c^	50.6 ^a,b,c^	46.8 ^a,b,c^	42.9	20.0
Antipsychotic (p < .0001)	46.3^a^	46.1^b^	51.1	59.4^c^	66.3 ^a,b^	68.2 ^a,b,c^	64.3	70.0
Benzodiazepine (p < .001)	2.1^a^	2.2^b^	4.6	3.9	16.1^a,b^	8.3	0	5.0
CNS stimulant (p < .0001)	18.0^a^	29.0^b^	34.1^c^	47.7^a,b^	28.8^d^	46.8^a,b,c,d^	42.9	65.0
Epileptic mood stabilizers (p < .0001)	7.4^a^	5.4	6	8.8^c^	16.7^b^	21.5^b^	1	1.4^a,b,c^
Other sedation	12.0	13.3	19.3	19.5	18.5	16.2	14.3	0
Number of psychotropic medications used (p < .0001)								
None	43.1	42.3	27.3	25.0	25.5	19.8	21.4	20.0
1	21.6	20.6	20.5	20.3	15.2	15.9	7.1	25.0
2	23.0	24.4	27.3	30.5	27.2	32.5	57.1	35.0
3 or more medications classes	12.4	12.7	25.0	24.2	32.1	31.9	14.3	20.0
Mean number of days on psychotropic medication	Mean							
Antidepressant (p < .0001)	51.7^a^	29.6^b^	86.6^b^	39.3^c^	122.5^a,b,c^	106.0^a,b,c^	62.0	43.0
Antipsychotic (p < .0001)	90.4^a^	106.4^b^	103.2	121.1^c^	191.3^a,b^	210.5^a,b,c^	141.6	221.9
Benzodiazepine (p < .001)	1.9^a^	2.3^b^	2.1	3.8	13.7^a,b^	7.4	0	1.4
CNS stimulant (p < .0001)	30.3^a^	60.9^b^	58.4^c^	114.1^a,b^	64.9^d^	124.5^a,b,c,d^	92.3	67.5
Epileptic mood stabilizers (p < .0001)	15.8^a^	14.1^b^	5.6^c^	5.1^d^	39.1^b^	36.8 ^b,d^	52.6	80.3^a,b,c,d^

Generalized Linear Models (GLMs) were used to identify significant differences in psychotropic medication use. Sidak correction to adjust for the multiple comparisons was used in the GLMs. Significant differences by intersectional sex and race/ethnicity are presented in the parentheses if p < .05. Pairwise significance between intersectional sex and race/ethnicity are noted with small letters. The same letter indicates significant difference between the intersectional groups at p < .05. The mean numbers of other sedation (other than epileptic mood stabilizers) use is not presented here as the percentages adolescents using other sedation did not differ by the intersectional sex and race/ethnicity at p < .05.

The most prescribed psychotropic medication was antipsychotics, followed by antidepressants and CNS stimulants. More than two thirds of white females and males and nearly two thirds of females and males in other racial/ethnic group were prescribed with antipsychotics. Mean number of days prescribed for antipsychotics were significantly higher for white females and males (191.3 days and 210.5 days, respectively) than those for African American females and males and Hispanic males (mean days ranging from 90.4 days to 121.1 days, p < .0001).

One half of white females, 46.8% of white males and 48.9% of Hispanic females were prescribed with antidepressants. The mean number of days prescribed for antidepressants was significantly higher for white females and males (122.5 and 106 days, respectively) than those for African American and Hispanic females and males (ranging from 29.6 to 86.6 days, p < .0001).

Benzodiazepines were the most prescribed among white females (16.1%) followed by white males (8.3%). The mean number of days prescribed for benzodiazepines was significantly higher for white females compared to those for African females and males (13.7 days vs. 1.9 - 2.3 days, p < .001).

Sixty-five percent of males in other racial/ethnic group, 47% of Hispanic males and white males, and 42.9% females in other race/ethnicity were prescribed with CNS stimulants. The mean number of days prescribed for CNS stimulants was the highest for white males (124.5 days vs. 30.3 days–114.1 days for other groups, p < .0001).

The percentages of adolescents prescribed for epileptic mood stabilizers significantly differed with white males prescribed the most (21.5%), followed by white females (16.7%), Hispanic males (8.8%), African American females (7.4%), African American males (5.4%), and other racial/ethnic females and males (1% and 1.4%, respectively). The mean number of days prescribed for epileptic mood stabilizers were significantly higher for other racial/ethnic males (80.3 days) than that for other groups (ranging from 5.1 to 39.1 days, p < .0001), except other racial/ethnic females. Other sedation use did not significantly differ by sex and race/ethnicity.

## Discussion

We found that a wide spectrum of psychiatric conditions that are commonly diagnosed in adolescents in the year prior to when they are first diagnosed with schizophrenia as previously reported in the literature (7, 8, 29). Over forty-five percent of the adolescents had one or more psychiatric diagnoses preceding schizophrenia. Previous research reports that the risk of developing schizophrenia is up to 4.4 times higher among adolescents who have been diagnosed with conduct disorder/ODD for five years or longer (30). Our study finds that at least one in every four African American males and females and males in other racial/ethnic group are diagnosed with conduct disorder/ODD before getting diagnosed with schizophrenia.

The prior psychiatric diagnoses may reflect the psychiatric comorbidities, but it may also be that psychosis or schizophrenia symptoms are misinterpreted in the presence of other symptoms and result in inappropriate labeling and treatment. For example, mood or psychotic symptoms are frequently associated with initial onset of bipolar disorder or schizophrenia in adolescence (25); thus, the chance of inappropriate labeling and treatment increases for these symptoms. The instability of first psychotic diagnoses given in children and adolescents should also be noted. Psychiatric diagnosis in adolescents are frequently revised during the subsequent course of treatment from affective psychosis or schizoaffective disorder to schizophrenia and vice versa as the adolescents and their illness mature. Careful screening and evaluation to validate diagnoses is important as the presence of certain psychiatric morbidity is common among people with schizophrenia during the prodromal period (31–35).

As found in previous studies, there were significant differences in psychiatric diagnoses by sex and race/ethnicity among adolescents. Significantly higher percentages of white males and females are diagnosed with bipolar disorder; significantly higher percentages of African American males and females are diagnosed with conduct/ODD; a significantly higher percentage of Hispanic females are diagnosed with depression; and higher percentages of African American and Hispanic females are diagnosed with PTSD.

Previous findings on differences in psychiatric diagnoses by sex and race/ethnicity have been limited to additive effects of sex and race/ethnicity. For example, although females are known to have higher prevalence of depression compared to males, little empirical evidence on the interplay of sex and race/ethnicity on depression exists. Different application of diagnostic criteria by clinicians, clinician bias against different sex and race/ethnicity, families' distrust in mental health professionals, and stigma associated with mental illness can result in misinterpretation of psychiatric symptoms and lead to inappropriate treatment of preceding psychiatric conditions and schizophrenia. Our study findings indicate the importance of further research on interplay of sex and race/ethnicity to gain in-depth insights into this disparity.

According to the data from the 2014 National Survey on Drug Use and Health, 21.3% of young adults aged 18–25 years with mental illness receive outpatient mental health services (36). Based on this report, we expected a similar level of outpatient therapy use for our study sample. The low use of outpatient therapy among the adolescents to treat their preceding psychiatric conditions is alarming. As the lack of outpatient therapy use can negatively affect symptom remission and recovery after getting diagnosed with schizophrenia (20–22), developing innovative ways to engage adolescents in outpatient therapy is important.

The high utilization of hospitalization and residential treatment care particularly, among African Americans and other race/ethnicity to treat their preceding psychiatric conditions confirms the documented disparity in mental health service use among adolescents. Individualized care to meet unique treatment need of adolescents by sex and race/ethnicity is important. Interventions such as the Recovery After an Initial Schizophrenia Episode (RAISE) and the Early Detection and Intervention for Prevention of Psychosis Program (EDIPPP) (21–24) that provide individualized specialty care coordination for psychosis or schizophrenia are currently available only by help-seeking or referral by health care professionals for individuals with first episode. To maximize the benefit of these interventions, there needs to be public policy to offer these individualized care coordination interventions at the population level. Additionally, developing innovative bridge or care connection programs that will smoothly connect the care between treatment for preceding psychiatric conditions and schizophrenia and a smooth transition from psychiatric hospitalization or residential treatment care to community-based care for adolescents is important.

Over 70% of the adolescents included in our study used one or more classes of psychotropic medications during one year prior to the first schizophrenia diagnosis. Considering that 45% of adolescents were diagnosed with preceding psychiatric disorders, it is likely that they also were prescribed with psychotropic medications from primary care physicians. Use of prescription medication for psychiatric disorders has increased among adolescents as evidenced in previous research (37). Using a nationally representative data, Anderson et al. (38) found that almost one-third of adolescents with outpatient visits for mental health conditions see primary care physicians and only a little over a quarter see psychiatrists. The study also reports that primary care physicians prescribe medications to a higher percentage of children with ADHD than did psychiatrists (73.7% vs. 61.4%) (38).

Our study reveals that more than one third of Hispanic adolescents, white males and adolescents in other racial/ethnic group are prescribed with CNS stimulants one year prior to their first schizophrenia diagnosis. Previous research reports that one in 660 adolescents and young adults with ADHD who receive prescription stimulants develop onset of psychosis (39). Our finding that CNS stimulants are commonly prescribed for the adolescents in the year prior to their being diagnosed with schizophrenia raises the question as to whether the prescription of stimulants may have increased the risk of schizophrenia as they are known to exacerbate psychotic symptoms (40).

For the early detection of psychosis, personalized psychosis risk prediction models have been developed and tested. For example, the North American Prodrome Longitudinal Study (NAPLS2) identifies clinical high-risk based on unusual thoughts, suspiciousness, verbal learning, symbol coding, social functioning decline, age, and family history (41). Integrating health claims data in the psychosis risk prediction models can lead to gaining in-depth insights into ideal timing of tailored interventions for adolescents with preceding psychiatric conditions.

Future research that analyzes temporal relationship between diagnoses given and different psychotropic medications prescribed can provide insights into practitioner understanding and behavior at the time of the diagnoses. The analysis also can provide more in-depth insights into the practical effects of the delay in making schizophrenia diagnosis or identifying a psychotic disorder at the time the preceding psychotic diagnoses were made. For example, our finding on the significantly higher percentage of adolescents with epileptic mood stabilizer prescriptions and the significantly higher percentage of adolescents with anxiety disorder diagnosis for other race/ethnic group compared to all other groups may indicate prescribers' behavior of reluctance to put a psychotic diagnosis into record of adolescents with certain race/ethnicity rather than failing to recognize that the adolescents were experiencing some psychosis.

Several study limitations should be mentioned. Our study included adolescents enrolled in Medicaid in Pennsylvania and thus, the interpretation of the study findings is limited to geographic areas with similar sociodemographic conditions. The preceding conditions were identified for one year prior and post the adolescents' first schizophrenia diagnosis. Due to the limited observation period, there is a chance that we have failed to identify certain conditions that tend to develop for longer term during adolescence. Medicaid populations can differ from non-Medicaid populations in their access to treatment. Thus, the study results cannot be generalized to the overall adolescent population. Finally, the Medicaid data we used is old and there may have been some changes in clinicians' diagnosis, prescription and treatment behaviors. Despite the limitations, our study findings provide much needed information on comprehensive psychiatric diagnoses and mental health treatment use preceding schizophrenia in adolescents and address important future directions for research, policy and practice.

## Conclusion

Careful screening and evaluation to validate diagnoses is important as the presence of certain psychiatric morbidity is common among people with schizophrenia during the prodromal period. Developing acceptable and accessible interventions that will prevent psychiatric hospitalization and residential treatment care and improve care coordination to smoothly connect to the care for schizophrenia is important to mitigate complexity in the treatment for adolescents and reduce cost burden for families and the society. Integrating health claims data in the development of schizophrenia risk conversion models can be useful in effectively predicting ideal timing of tailored interventions for adolescents with preceding psychiatric conditions.

## Data Availability Statement

The datasets generated for this study will not be made publicly available. The data license agreement restricts the access only to the University of Pennsylvania Center for Mental Health.

## Ethics Statement

The studies involving human participants were reviewed and approved by the University of Pennsylvania Institutional Review Board. Written informed consent from the participants' legal guardian/next of kin was not required to participate in this study in accordance with the national legislation and the institutional requirements.

## Author Contributions

CK-Y led conceptual design of the study, literature review, data analysis, interpretation of the study results, and manuscript writing. BC and ST contributed to literature review and manuscript writing. JL contributed to interpretation of the study results and manuscript writing. DM contributed to conceptual design of the study, interpretation of the study results and manuscript writing. Y-LW contributed to conceptual design of the study and manuscript writing. CE provided clinical expertise and contributed to conceptual design of the study and manuscript writing.

## Funding

This Study was funded through the National Institute of Health's Training in Sex and Gender Differences Research to Imrpove Womens' Health program (Grant number: 5-K12-HD-085848-04).

## Conflict of Interest

The authors declare that the research was conducted in the absence of any commercial or financial relationships that could be construed as a potential conflict of interest.
